# Elastic mechanics solution of thermal expansion of bi-material curved beam and its application to negative thermal expansion metamaterials

**DOI:** 10.1038/s41598-022-16036-2

**Published:** 2022-07-11

**Authors:** Jingxiang Huang, Minghui Fu, Binbin Zheng

**Affiliations:** 1 Department of Applied Mechanics and Engineering, Sun Yat-Sen University, Guangzhou, 510275 People’s Republic of China; 2 School of Mechatronic Engineering, Guangdong Polytechnic Normal University, Guangzhou, 510665 People’s Republic of China

**Keywords:** Composites, Mechanical properties

## Abstract

Thermal stress impacts various engineering fields significantly, such as aerospace and precision instruments. This adverse effect can be greatly reduced, if not eliminated, by the application of micro-thermal expansion materials, and bi-material beams are widely utilized in the design of micro-thermal expansion structures, thereby exhibiting great application potentials. The elasticity solution of bi-material curved beam under free thermal expansion has been proposed by scholars. Based on this solution, the simplified form is proposed in this paper, and extended to the case where the rotation angles at both ends of the circular arc are constrained under thermal loads. Besides, the geometric parameters and the nonlinear problems of the thermal expansion of bi-material curved beam are analyzed. In addition, a novel type of negative thermal expansion material has been designed by applying the bi-material curved beam to the tetra chiral and anti-tetra chiral materials. The proposed material has greater negative thermal expansion effect than the traditional tetra and anti-tetra chiral materials that are with straight beams.

## Introduction

The coefficient of thermal expansion (CTE) is an important indicator of the thermophysical properties of materials and is used to characterize geometric variations caused by temperature changes. Most natural materials have positive CTEs that expand when heated and shrink when cooled and they are called positive thermal expansion (PTE) materials. However, some materials have extremely small or even negative CTEs and they are called microthermal expansion materials and negative thermal expansion (NTE) materials. For example, some zeolites are NTE materials^1^. In engineering fields such as aerospace and precision instruments, thermal stress or expansion caused by ambient temperature change has affected structural safety or instrument accuracy. Therefore, micro-thermal expansion materials, which can alleviate these problems, exhibit high application potential^2^.

An intuitive design for microthermal expansion materials is mixing PTE and NTE materials in an appropriate proportion^3^. However, natural NTE materials are rare and their NTE effect can only be observed in narrow temperature ranges that are either very low or high^4^. More significantly, natural NTE materials tend to have significant anisotropy and low mechanical strength, which are unsuitable for engineering applications^5,6^. This led to the studies of NTE materials focusing on the composition of two or more PTE materials, the advantage of which is that CTEs can be flexibly controlled by meso-structural design.

According to the thermal expansion deformation mechanism of the material lattice, NTE metamaterials can be divided as ones that are stretch-dominant and ones that are bending-dominant^2^. The former achieves the NTE effect via axial deformation. Examples include planar structures based on the bi-material triangle^7–12^, spatial structures based on the bi-material tetrahedron^13,14^, cylindrical shell structures^15^, and three-dimensional structures based on the bi-material triangles^16,17^. These tend to exhibit high relative stiffness, but they have a limited regulation range of CTE. The latter includes ones that mainly achieves the NTE effect via the bending of bi-material straight beams or curved beams when heated. Examples include chiral structures^18,19^, anti-chiral structures^19–21^, and re-entrant structures^22^. These are essentially some of the auxetic materials with beams being replaced by bi-material beams. Also, some bending-dominant NTE structures consist of two kinds of single-material beams. Examples include star-shaped structures with reinforcing rods and their three-dimensional extension^23,24^, re-entrant hexagonal structures with reinforcing rods^25^, and other novel re-entrant structures^26^. It is worth noting that bi-material beams are widely used in the bending-dominant NTE materials and these materials usually have high flexibility, a wide range of adjustment of CTEs, and they can simultaneously achieve NTE and negative Poisson's ratio (NPR)^20^. Therefore, bi-material beams are greatly significant for manufacturing intelligent sensors and realizing structural multi-functional integration. With the development of the fabrication methods of these metamaterials such as 3D printing, experimental validation of the properties of these materials became reality which promoted the development of research in this field^27,28^.

Timoshenko proposed the analytical formula of curvature^29^ in 1925 for the thermal expansion of bi-material straight beams and this formula has been widely used^30^. The formula also applies to bi-material circular beams, except that curvature needs to be replaced by curvature increment^31^. This conclusion has also been widely used^20,31,32^. Ni et al. covered the actuation material on the frame beam to form a bi-material beam and studied its thermal expansion^33^. The result can be the same as the formula in Ref^31^ when the coverage is 100%. However, the analysis in the current study shows that there exists large error between the formula in Ref^31^ and the numerical simulation results, and wrong results can even occur in some cases when the center angle of the arc is small (< 80°) or when the ratio of the beam thickness to the beam radius exceeds 1/10. For example, positive value of CTE may be calculated as negative for certain center angles. Therefore, it is necessary to propose a more accurate theory to study the thermal deformation of bi-material circular beams. In 2019, Gonczi proposed the elasticity method of thermal expansion bending of bi-material circular arc curved beam with the utility of the theory of elastic small deformation, and verified its validity through numerical simulation results^34^. However, there was a lack of further parameter analysis on the thermal expansion effect and other properties of circular arc.

In this paper, based on the idea in Ref^34^, the elasticity solution for thermal expansion bending of bi-material circular arc curved beam was proposed, and the result was simplified with almost no influence on the accuracy. Based on this result, the CTE of the bi-material circular arc was studied and the finite element result shows that this formula has a very high accuracy that no matter how small the central angle is or how large the ratio of thickness to radius is, it is almost identical with the finite element result. Therefore, it can be better applied to the parameter analysis and optimization design of flexible NTE structures composed of bi-material circular arc curved beam. In addition, the analytical results in Ref^34^ are extended to the case where the rotation angles at both ends of the circular arc are constrained, and the geometric parameters and the nonlinear problems of thermal expansion of bi-material curved beam are analyzed. Besides, two novel NTE metamaterials are designed by applying the bi-material circular arc to the tetra chiral/anti-tetra chiral honeycomb. Parameter analyses show that the proposed metamaterials could obtain higher NTE values compared with the NTE metamaterials proposed earlier by Yu et al.^19^.

## Derivation, simplification and verification of analytical formulas

### Pure bending of a single-layer curved beam under thermal–mechanical coupling

**Figure 1:**
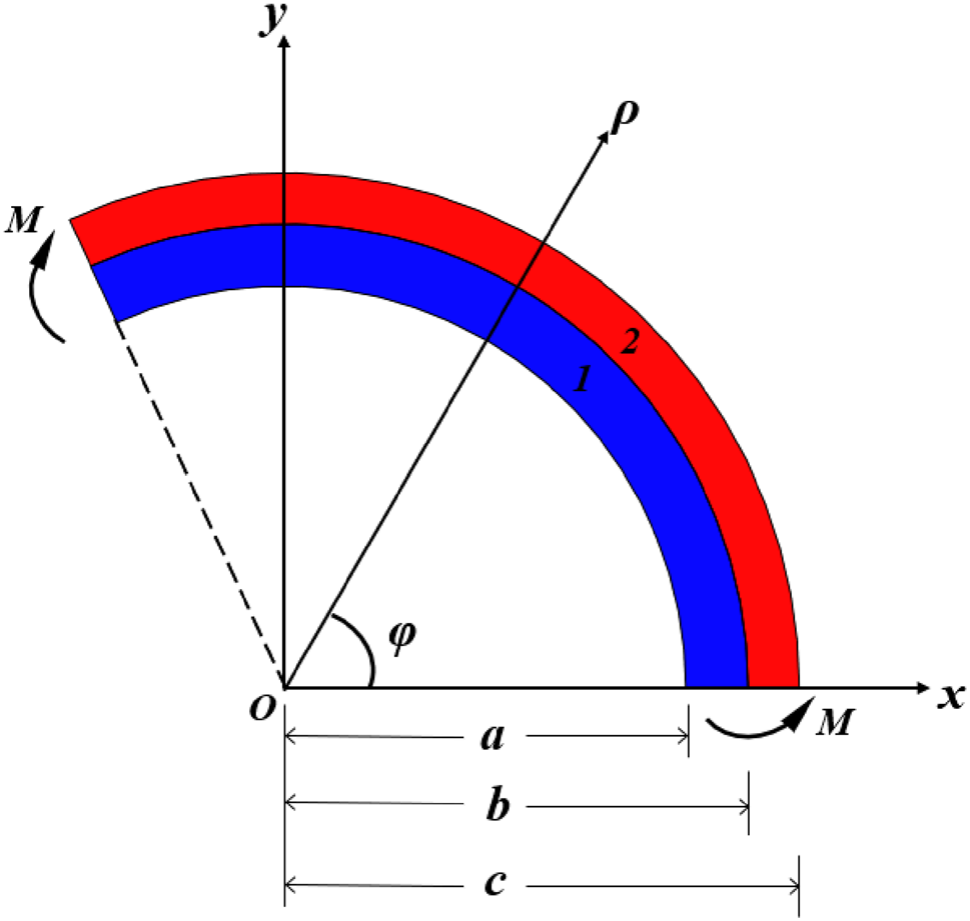
Pure bending of the curved beam under the action of force couple and temperature variation.

Figure 1 shows a curved beam subjected to the equal bending moment $M$ at the two ends and a homogeneous temperature load $\Delta T$, where $a$, $b$ and $c$ represent the inner radius of blue region, the radius of the boundary between blue region and red region, and the outer radius of red region, respectively. $\varphi$ represents the polar angle in polar coordinates. The curvature center $O$ is defined as the coordinate origin, and the polar angle is measured counterclockwise from the *x*-axis of the curved beam. Based on the theory of elastic small deformation, Gonczi proposed the stress field and displacement field inside the curved beam, and the boundary condition when there is no bending moments at both ends of the curved beam^34^.

For material *i* ($$i = 1,2$$):

1 $$\left\{ {\begin{array}{*{20}l} {\sigma_{\rho i} = \frac{{A_{i} }}{{\rho^{2} }} + B_{i} \ln \rho + C_{i} } \hfill \\ {\sigma_{\varphi i} = - \frac{{A_{i} }}{{\rho^{2} }} + B_{i} \left( {1 + \ln \rho } \right) + C_{i} } \hfill \\ {u_{\rho i} = \frac{1}{{E_{i} }}\left[ { - \left( {1 + \mu_{i} } \right)\frac{{A_{i} }}{\rho } - B_{i} \rho + \left( {1 - \mu_{i} } \right)\left( {B_{i} \ln \rho + C_{i} } \right)\rho } \right]} \hfill \\ {\quad \quad + \alpha_{i} \Delta T\rho + D_{1} \cos \varphi + D_{2} \sin \varphi } \hfill \\ {u_{\varphi i} = \frac{{2B_{i} \rho \varphi }}{{E_{i} }} + D_{1} \sin \varphi - D_{2} \cos \varphi + D_{3} \rho } \hfill \\ \end{array} } \right.$$

where $A_{i} ,B_{i} ,C_{i} ,D_{1} ,D_{2} ,D_{3}$ are the undetermined coefficients, $\sigma_{\rho i} ,\sigma_{\varphi i}$ represent the normal stress and the circumferential stress of material *i*, $u_{\rho i} ,u_{\varphi i}$ represent the normal displacement and the circumferential displacement of material *i*, respectively. $\rho$ and $\varphi$ represent the polar radius and the polar angle in the polar coordinates.$E_{i} ,\mu_{i} ,\alpha_{i}$ represent the modulus, Poisson’s ratio and CTE of material *i*, respectively. $\Delta T$ represents the change of temperature. It should be mentioned that the blue and red regions represent material 1 and 2, respectively.

The boundary conditions are as follows:

2 $$\left\{ {\begin{array}{*{20}l} {\left. {\sigma_{{\rho {1}}} } \right|_{\rho = a} = 0} \hfill \\ {\left. {\sigma_{\rho 2} } \right|_{\rho = c} = 0} \hfill \\ {\int_{a}^{b} {\rho \sigma_{\varphi 1} {\text{d}}\rho + } \int_{b}^{c} {\rho \sigma_{\varphi 2} {\text{d}}\rho } = 0} \hfill \\ {\left. {\sigma_{{\rho {1}}} } \right|_{\rho = b} = \left. {\sigma_{\rho 2} } \right|_{\rho = b} } \hfill \\ {\left. {u_{{\rho {1}}} } \right|_{\rho = b} = \left. {u_{\rho 2} } \right|_{\rho = b} } \hfill \\ {\left. {u_{{\varphi {1}}} } \right|_{\rho = b} = \left. {u_{\varphi 2} } \right|_{\rho = b} } \hfill \\ \end{array} } \right.$$

In Eq. (1), $D_{1} ,D_{2} ,D_{3}$ represent the rigid body displacements and rotations of a circular arc, and they are regarded as arbitrary constants without affecting the stress and strain field of the circular arc. Therefore, if the center of the circular arc is taken as the reference point, it can be set as $D_{1} = D_{2} = D_{3} = 0$. In this way, the number of undetermined coefficients in Eq. (1) is equal to the number of equations in Eq. (2), and the equations are solvable:

3 $$\left\{ {\begin{array}{*{20}l} {A_{1} = \frac{{\left( {\alpha_{1} - \alpha_{2} } \right)a^{2} b^{2} E_{1} E_{2} \Delta T}}{N} \cdot \left[ {j\left( {gE_{1} - jE_{2} } \right) - 4b^{2} c^{2} \ln \frac{c}{b}\left( {E_{1} \ln \frac{a}{b} - E_{2} \ln \frac{c}{b}} \right)} \right]} \hfill \\ {B_{1} = \frac{{4\left( {\alpha_{1} - \alpha_{2} } \right)b^{2} E_{1}^{2} E_{2} \Delta T}}{N} \cdot \left( {ja^{2} \ln \frac{a}{b} - gc^{2} \ln \frac{c}{b}} \right)} \hfill \\ {C_{1} = - B_{1} \ln b + \frac{{\left( {\alpha_{1} - \alpha_{2} } \right)b^{2} E_{1} E_{2} \Delta T}}{N} \cdot \left[ { - j\left( {gE_{1} - jE_{2} + 4a^{2} E_{1} \ln^{2} \frac{a}{b}} \right)} \right.} \hfill \\ {\quad \quad \left. { + 2c^{2} \ln \frac{c}{b}\left( {2a^{2} E_{1} \ln \frac{a}{b} - 2b^{2} E_{2} \ln \frac{c}{b}} \right)} \right]} \hfill \\ \end{array} } \right.$$

where

4 $$\left\{ {\begin{array}{*{20}l} {N = \left( {gE_{1} - jE_{2} } \right)\left\{ {b^{4} \left( {m - p} \right) + a^{2} c^{2} \left( {n - q} \right) + b^{2} \left[ {a^{2} \left( {p - n} \right) + c^{2} \left( {q - m} \right)} \right]} \right\}} \hfill \\ {\quad \quad + 4b^{2} \left\{ {a^{2} E_{1} \left[ {b^{2} \left( {m - p} \right) + c^{2} \left( {q - n} \right)} \right]\ln^{2} \frac{a}{b}} \right.} \hfill \\ {\left. {\quad \quad + c^{2} E_{2} \left[ {b^{2} \left( {p - m} \right) + a^{2} \left( {n - q} \right)} \right]\ln^{2} \frac{c}{b} + 2a^{2} c^{2} E_{1} E_{2} \ln^{2} \frac{a}{c}} \right\}} \hfill \\ {g = a^{2} - b^{2} } \hfill \\ {j = c^{2} - b^{2} } \hfill \\ {m = \left( {\mu_{1} - 1} \right)E_{2} } \hfill \\ {n = \left( {\mu_{1} + 1} \right)E_{2} } \hfill \\ {p = \left( {\mu_{2} - 1} \right)E_{1} } \hfill \\ {q = \left( {\mu_{2} + 1} \right)E_{1} } \hfill \\ \end{array} } \right.$$

$A_{2} , \, B_{2} , \, C_{2}$ can be obtained by interchanging material parameters and $a, \, c$ in $A_{1} , \, B_{1} , \, C_{1}$.

By substituting Eq. (3) into Eq. (1), the displacement component at $\rho = b$ can be obtained:

5 $$\left\{ {\begin{array}{*{20}l} {\left. {u_{\rho 1} } \right|_{\rho = b} = \frac{1}{{E_{{1}} }}\left[ { - \left( {1 + \mu_{{1}} } \right)\frac{{A_{{1}} }}{b} - B_{1} b + \left( {1 - \mu_{{1}} } \right)\left( {B_{{1}} \ln b + C_{{1}} } \right)b} \right] + \alpha_{{1}} \Delta Tb} \hfill \\ {\left. {u_{{\varphi {1}}} } \right|_{\rho = b} = \frac{{2B_{{1}} b\varphi }}{{E_{{1}} }}} \hfill \\ \end{array} } \right.$$

**Figure 2:**
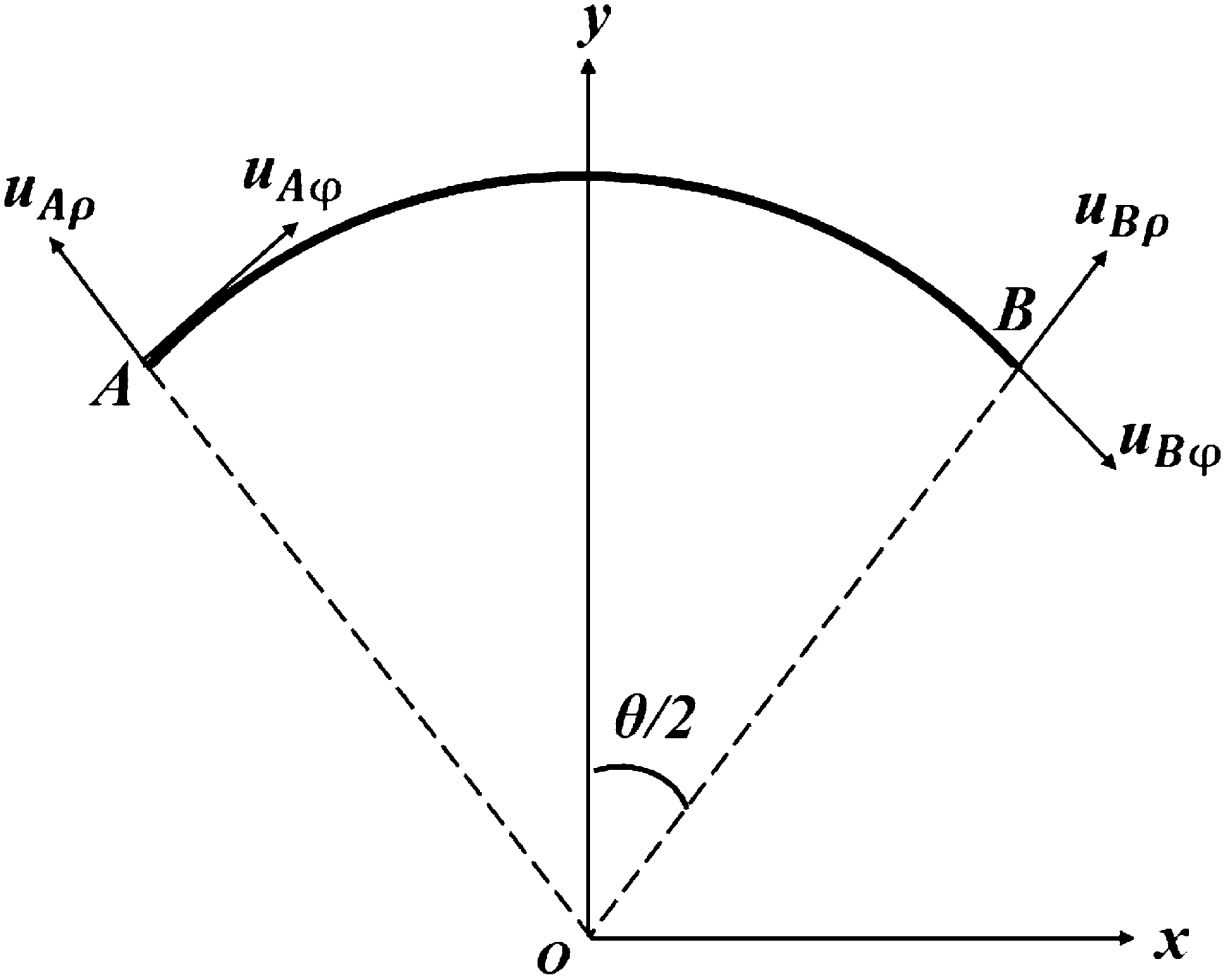
Deformations at both ends of the curved beam.

Figure 2 shows the schematic diagram of the deformations at both ends of a curved beam. According to the geometric relationship, the relative deformation at both ends of the curved beam is:

6 $$\begin{aligned} \Delta u_{x} & = \left( {u_{B\varphi } - u_{A\varphi } } \right)\cos \frac{\theta }{2} + \left( {u_{B\rho } + u_{A\rho } } \right)\sin \frac{\theta }{2} \\ & = \frac{{2B_{{1}} b\theta }}{{E_{{1}} }}\cos \frac{\theta }{2} + 2\sin \frac{\theta }{2}\left. {u_{\rho 1} } \right|_{\rho = b} \\ \end{aligned}$$

In this way, the equivalent CTE of the curved beam can be obtained:

7 $$\begin{aligned} \overline{\alpha } & = \frac{{\Delta u_{x} }}{{2b\sin \frac{\theta }{2}\Delta T}} \\ & = \frac{1}{{E_{{1}} \Delta T}}\left[ { - \left( {1 + \mu_{{1}} } \right)\frac{{A_{{1}} }}{{b^{2} }} - B_{1} + \left( {1 - \mu_{{1}} } \right)\left( {B_{{1}} \ln b + C_{{1}} } \right)} \right] + \frac{{B_{{1}} \theta }}{{E_{{1}} \Delta T}}\cot \frac{\theta }{2}{ + }\alpha_{{1}} \\ \end{aligned}$$

where $A_{1} , \, B_{1} , \, C_{1}$ are given by Eq. (3).

### Formula simplification

The study in Ref^34^ proposed only the solving method of Eq. (3) but did not show the final results, the reason of which may be that the results are too complex. To solve this problem, in this section, Eq. (7) is simplified by introducing small quantity hypothesis which makes the formula more applicable. ${{h_{1} } \mathord{\left/ {\vphantom {{h_{1} } b}} \right. \kern-\nulldelimiterspace} b}$ and ${{h_{2} } \mathord{\left/ {\vphantom {{h_{2} } b}} \right. \kern-\nulldelimiterspace} b}$ are usually small for beams. Assuming that:

8 $$\left\{ {\begin{array}{*{20}l} {h_{1} = b - a} \hfill \\ {h_{2} = c - b} \hfill \\ {\ln \frac{a}{b} = - \frac{{h_{1} }}{b} - \frac{1}{2}\left( {\frac{{h_{1} }}{b}} \right)^{2} - \frac{1}{3}\left( {\frac{{h_{1} }}{b}} \right)^{3} } \hfill \\ {\ln \frac{c}{b} = \frac{{h_{2} }}{b} - \frac{1}{2}\left( {\frac{{h_{2} }}{b}} \right)^{2} + \frac{1}{3}\left( {\frac{{h_{2} }}{b}} \right)^{3} } \hfill \\ \end{array} } \right.$$

Substitute Eq. (8) into Eq. (3) and remove the higher-order terms of ${h \mathord{\left/ {\vphantom {h b}} \right. \kern-\nulldelimiterspace} b}$, the following is obtained:

9 $$\left\{ {\begin{array}{*{20}l} {A_{1} = \frac{{4\left( {\alpha_{1} - \alpha_{2} } \right)b^{4} h_{2} E_{1} E_{2} \Delta T}}{3N} \cdot \left[ { - E_{2} h_{2}^{3} + 3bE_{1} h_{1} \left( {h_{1} + h_{2} } \right)} \right.} \hfill \\ {\quad \quad \left. { + E_{1} h_{1} \left( { - 5h_{1}^{2} - 3h_{1} h_{2} + h_{2}^{2} } \right)} \right]} \hfill \\ {B_{1} = \frac{{8\left( {\alpha_{1} - \alpha_{2} } \right)b^{2} h_{1} h_{2} E_{1}^{2} E_{2} \Delta T}}{3N} \cdot \left( {3b - h_{1} + h_{2} } \right)\left( {h_{1} + h_{2} } \right)} \hfill \\ {C_{1} = - B_{1} \ln b + \frac{{4\left( {\alpha_{1} - \alpha_{2} } \right)b^{2} E_{1} E_{2} \Delta T}}{3N} \cdot \left( { - 3bE_{1} h_{1}^{2} h_{2} + 5E_{1} h_{1}^{3} h_{2} - 3bE_{1} h_{1} h_{2}^{2} } \right.} \hfill \\ {\quad \quad \left. { + 3E_{1} h_{1}^{2} h_{2}^{2} - E_{1} h_{1} h_{2}^{3} + E_{2} h_{2}^{4} } \right)} \hfill \\ {N = - \frac{8}{3}b^{2} \left[ {E_{1}^{2} h_{1}^{4} + E_{2}^{2} h_{2}^{4} + 2E_{1} E_{2} h_{1} h_{2} \left( {2h_{1}^{2} + 3h_{1} h_{2} + 2h_{2}^{2} } \right)} \right]} \hfill \\ \end{array} } \right.$$

By Substituting Eq. (9) into Eq. (7), the following is obtained:

10 $$\begin{aligned} \overline{\alpha } & = \alpha_{1} + \frac{\begin{gathered} E_{2} h_{2} \left( {\alpha_{1} - \alpha_{2} } \right)\left[ { - E_{2} h_{2}^{3} + 6E_{1} bh_{1} \left( {h_{1} + h_{2} } \right) + E_{1} h_{1} \left( { - 6h_{1}^{2} - 3h_{1} h_{2} + 2h_{2}^{2} } \right)} \right. \hfill \\ \left. { \, - E_{1} h_{1} \left( {3b - h_{1} + h_{2} } \right)\left( {h_{1} + h_{2} } \right)\theta \cot \left( {{\theta \mathord{\left/ {\vphantom {\theta 2}} \right. \kern-\nulldelimiterspace} 2}} \right)} \right] \hfill \\ \end{gathered} }{\begin{gathered} E_{1}^{2} h_{1}^{4} + E_{2}^{2} h_{2}^{4} + 2E_{1} E_{2} h_{1} h_{2} \left( {2h_{1}^{2} + 3h_{1} h_{2} + 2h_{2}^{2} } \right) \hfill \\ \hfill \\ \end{gathered} } \\ & = \alpha_{1} + \frac{\begin{gathered} \left( {\alpha_{1} - \alpha_{2} } \right)\left\{ { - 1 + 6\frac{{E_{1} h_{1} b}}{{E_{2} h_{2} h}}\left( {\frac{{h_{1} }}{{h_{2} }} + 1} \right)^{2} + \frac{{E_{1} h_{1} }}{{E_{2} h_{2} }}\left[ { - 6\left( {\frac{{h_{1} }}{{h_{2} }}} \right)^{2} - 3\frac{{h_{1} }}{{h_{2} }} + 2} \right]} \right. \hfill \\ \, \left. { - \frac{{E_{1} h_{1} }}{{E_{2} h_{2} }}\left[ {3\frac{b}{h}\left( {\frac{{h_{1} }}{{h_{2} }} + 1} \right) - \frac{{h_{1} }}{{h_{2} }} + 1} \right]\left( {\frac{{h_{1} }}{{h_{2} }} + 1} \right)\theta \cot \frac{\theta }{2}} \right\} \hfill \\ \end{gathered} }{{\left( {\frac{{E_{1} h_{1}^{2} }}{{E_{2} h_{2}^{2} }}} \right)^{2} + 1 + 2\frac{{E_{1} h_{1} }}{{E_{2} h_{2} }}\left[ {2\left( {\frac{{h_{1} }}{{h_{2} }}} \right)^{2} + 3\frac{{h_{1} }}{{h_{2} }} + 2} \right]}} \\ \end{aligned}$$

Equation (9) does not involve Poisson's ratio $\mu_{1}$ and $\mu_{2}$, as in the expansion of Eq. (3) after substituting Eq. (8) into it, the terms involving $\mu_{1}$ and $\mu_{2}$ are all higher-order terms of ${h \mathord{\left/ {\vphantom {h b}} \right. \kern-\nulldelimiterspace} b}$, so they are eliminated. This indicates that the Poisson's ratio of the base material has a negligible effect on the bi-material beam CTE. In fact, further calculation confirms that the numerical results of simplified formula Eq. (10) are almost identical with those of the original analytical formula Eq. (7). Therefore, considering $\mu_{1}$ and $\mu_{2}$ or not does not affect the NTE and NPR of the bi-material beam.

When $h_{1} = h_{2} = {h \mathord{\left/ {\vphantom {h 2}} \right. \kern-\nulldelimiterspace} 2}$, Eq. (10) can be further simplified to

11 $$\begin{aligned} \overline{\alpha } & = \frac{{24E_{1} E_{2} b\left( {\alpha_{1} - \alpha_{2} } \right)\left( {1 - \varphi \cot \varphi } \right) + h\left[ {E_{1}^{2} \alpha_{1} + E_{2}^{2} \alpha_{2} + 7E_{1} E_{2} \left( {\alpha_{1} + \alpha_{2} } \right)} \right]}}{{\left( {E_{1}^{2} + 14E_{1} E_{2} + E_{2}^{2} } \right)h}} \\ & = \frac{{{24}\frac{{E_{1} }}{{E_{2} }}\left( {\alpha_{1} - \alpha_{2} } \right)\left( {1 - \varphi \cot \varphi } \right) + \frac{h}{b}\left[ {\left( {\frac{{E_{1} }}{{E_{2} }}} \right)^{2} \alpha_{1} + \alpha_{2} + 7\frac{{E_{1} }}{{E_{2} }}\left( {\alpha_{1} + \alpha_{2} } \right)} \right]}}{{\frac{h}{b}\left[ {\left( {\frac{{E_{1} }}{{E_{2} }}} \right)^{2} + 14\frac{{E_{1} }}{{E_{2} }} + 1} \right]}} \\ \end{aligned}$$

### Validation of effectiveness

In 1996, Lakes derived the analytical formula of the CTE of bi-material curved beam as follows utilizing curvature increment instead of curvature in Timoshenko’s analytical formula for the thermal expansion of bi-material straight beams^31^:

12 $$\overline{\alpha }_{{{\text{Lakes}}}} = \frac{b \cdot \theta }{h} \times \frac{{6\left( {\alpha_{2} - \alpha_{1} } \right)\left( {1 + \frac{{h_{1} }}{{h_{2} }}} \right)^{2} }}{{3\left( {1 + \frac{{h_{1} }}{{h_{2} }}} \right)^{2} + \left( {1 + \frac{{h_{1} }}{{h_{2} }}\frac{{E_{1} }}{{E_{2} }}} \right)\left[ {\left( {\frac{{h_{1} }}{{h_{2} }}} \right)^{2} + \frac{{h_{1} }}{{h_{2} }}\frac{{E_{2} }}{{E_{1} }}} \right]}} \times \left( {\frac{1}{2}\cot \frac{\theta }{2} - \frac{1}{\theta }} \right)$$

In this section, we will use the finite element method to compare the accuracy and scope of application of the derived simplified formula and the formula in Ref^31^ [Eq. (12)].

**Figure 3:**
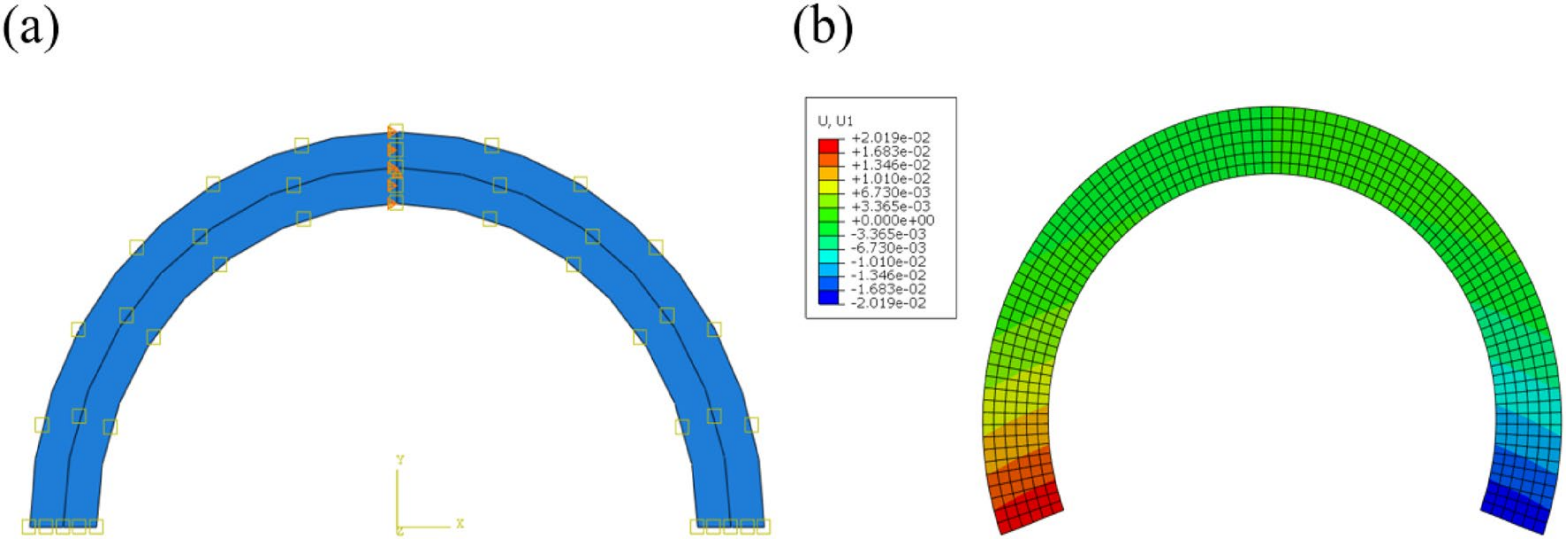
Finite element model: (**a**) initial state; (**b**) contour plots of displacement along *x* direction.

The 8-node plane stress element CPS8R of the finite element software Abaqus was utilized. Take $\theta = 180^{ \circ }$ as an example of numerical verification. Figure 3a shows the finite element model. The distance between the interface of the two materials and the center of the circle is $b = 80\;{\text{mm}}$, and the thicknesses of the two materials are $h_{1} = h_{2} = 8\;{\text{mm}}$. The two materials are PI ($E_{1} = 2.5\;{\text{Gpa}}$, $\mu_{{1}} { = 0}{\text{.34}}$, $\alpha_{{1}} = {{30\;{\text{ppm}}} \mathord{\left/ {\vphantom {{30\;{\text{ppm}}} {^\circ {\text{C}}}}} \right. \kern-\nulldelimiterspace} {^\circ {\text{C}}}}$) and PMMA ($E_{2} = {2}{\text{.5}}\;{\text{Gpa}}$, $\mu_{{2}} = {0}{\text{.3}}$, $\alpha_{{2}} = {{{7}0\;{\text{ppm}}} \mathord{\left/ {\vphantom {{{7}0\;{\text{ppm}}} {^\circ {\text{C}}}}} \right. \kern-\nulldelimiterspace} {^\circ {\text{C}}}}$), as mentioned in previous studies^33^. The upper part represents Material 1 and the lower part represents Material 2, with a grid of 100 (circumferential) × 6 (radial) and geometric nonlinearity is not considered in this section. After testing, the calculation results converge under this density of grids.

The middle section of arc is fixed and a temperature load of 1 °C is applied to obtain the equivalent CTE. Figure 3b shows the deformation of the beam. Then, the accuracy of the obtained analytical solution is verified by checking the error of the analytical solution and the numerical simulation when ${h \mathord{\left/ {\vphantom {h b}} \right. \kern-\nulldelimiterspace} b}$ changes.

**Figure 4:**
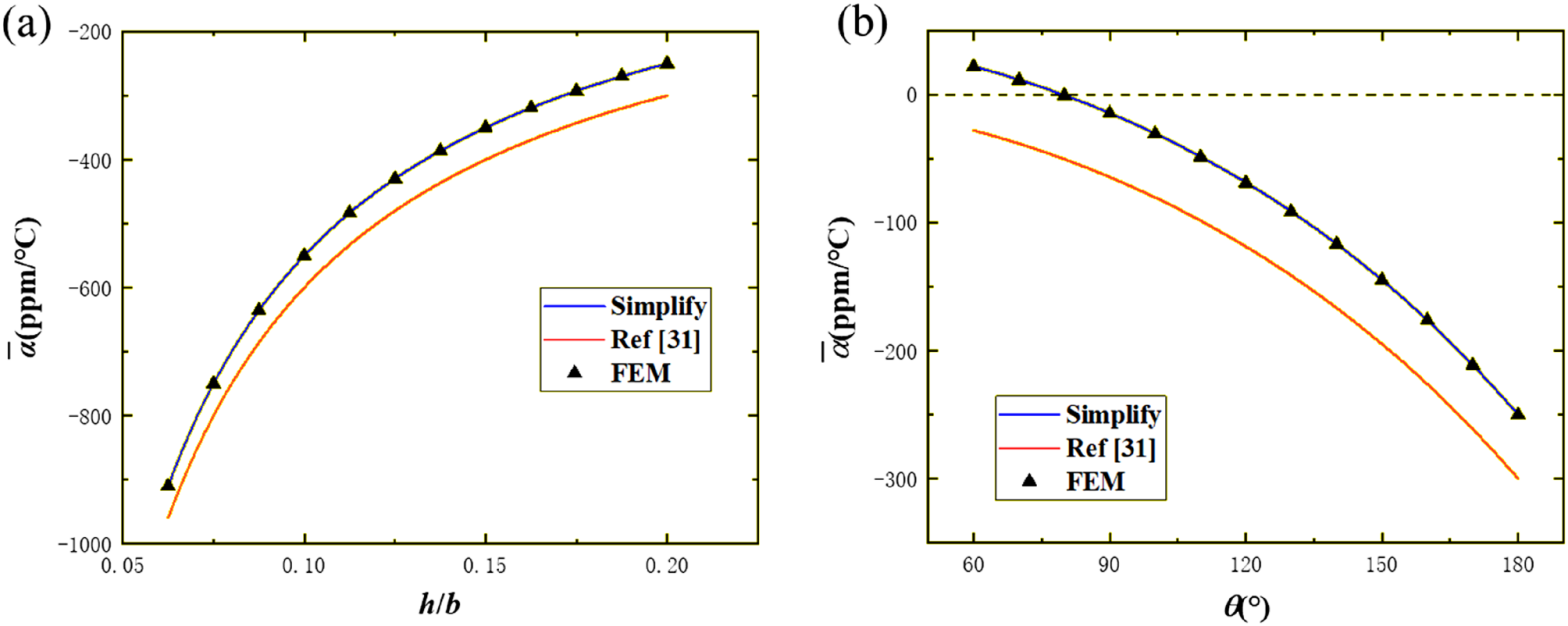
Results of the simplified formula, Ref^31^, and finite element method: (**a**) Equivalent CTE versus ${h \mathord{\left/ {\vphantom {h b}} \right. \kern-\nulldelimiterspace} b}$ at $b = 80\;{\text{mm}}$ and $\theta = 180^\circ$; (**b**) Equivalent CTE versus $\theta$ at $b = 80\;{\text{mm}}$ and $h = 16\;{\text{mm}}$.

With the thicknesses of two layers being equal ($h_{1} = h_{2} = {h \mathord{\left/ {\vphantom {h 2}} \right. \kern-\nulldelimiterspace} 2}$ and $\theta = 180^{ \circ }$), the accuracy of the proposed simplified formula and Eq. (12) as ${h \mathord{\left/ {\vphantom {h b}} \right. \kern-\nulldelimiterspace} b}$ varied is investigated. Figure 4a shows the equivalent CTE $\overline{\alpha }$ calculated by the proposed simplified analytical method (Simplify, blue curve), Eq. (12) (Ref^31^, red curve), and the finite element method (FEM, scatters) when ${h \mathord{\left/ {\vphantom {h b}} \right. \kern-\nulldelimiterspace} b}$ increases from 1/16 (i.e. $h = 5{\text{ mm}}$) to 1/5 (i.e. $h = 16{\text{ mm}}$). Further study revealed that the relative error of results calculated via Eq. (12) remains at around 5.5% while the results calculated via the proposed simplified analytical formula are highly consistent with numerical simulation results and the relative error remains at around 0, which indicates that the result is indeed the elasticity solution of the bi-material curved beam. It is worth noting that here, the relative error at a certain ${h \mathord{\left/ {\vphantom {h b}} \right. \kern-\nulldelimiterspace} b}$ is defined as the absolute value of the difference between the results calculated via the analytical formula and the numerical simulation results divided by the largest absolute value of the FEM results. Therefore, the proposed simplified analytical formula can be utilized to obtain the equivalent CTEs of complex structures based on bi-material curved beams.

Figure 4b shows the applicable scopes of the proposed simplified formula and Eq. (12) at ${h \mathord{\left/ {\vphantom {h {\left( {b\theta } \right)}}} \right. \kern-\nulldelimiterspace} {\left( {b\theta } \right)}} = {{1} \mathord{\left/ {\vphantom {{1} {10}}} \right. \kern-\nulldelimiterspace} {10}}$, that is, the slenderness ratio of the arc is 0.1. When $\theta$ is small (< 54°), numerical simulation results show that the $\overline{\alpha }$ of the curved beam is positive, while the result of Eq. (12) is negative. Besides, for any $\theta$, the relative error of results calculated via Eq. (12) remains at around 20% while the results calculated via the proposed simplified analytical formula are highly consistent with numerical simulation results and the relative error remains at around 0, which not only indicates that small $\theta$ is not the applicable scope of Eq. (12), but the accuracy of the proposed simplified formula is very high. This proves the advantage of the proposed simplified formula.

## Discussions

### Parameter analysis

First, the effect of the thickness of curved beam on its equivalent CTE is investigated. Figure 4a shows that the curved beam exhibits negative CTE, while NTE effect degrades as ${h \mathord{\left/ {\vphantom {h b}} \right. \kern-\nulldelimiterspace} b}$ increases. This can be attributed to the increase of the flexural rigidity of the curved beam with the increase of its thickness. As ${h \mathord{\left/ {\vphantom {h b}} \right. \kern-\nulldelimiterspace} b}$ increases, the curved beam is more difficult to bend, thereby its NTE effect is affected. Meanwhile, the absolute value of CTE ranges from 250–910 ppm/°C, which is an order of magnitude higher than that of component materials, specifically in the range of ${h \mathord{\left/ {\vphantom {h b}} \right. \kern-\nulldelimiterspace} b}$ shown in Fig. 4a. Therefore, bi-material curved beams can not only achieve NTE but also magnify the sensitivity of thermal expansion.

Then, the effect of the central angle $\theta$ of curved beam on its equivalent CTE is investigated. Figure 4b shows the equivalent CTE of the curved beam decreases monotonously from positive values to negative values as $\theta$ increases from 40° to 180°. This can be attributed to the increase of the rotation angles at both ends of the curved beam during thermal deformation with the increase of radian, which leads to a larger NTE. Therefore, according to the analyses above, ${h \mathord{\left/ {\vphantom {h b}} \right. \kern-\nulldelimiterspace} b}$ shall be minimized and $\theta$ shall be maximized to achieve the maximum NTE effect.

**Figure 5:**
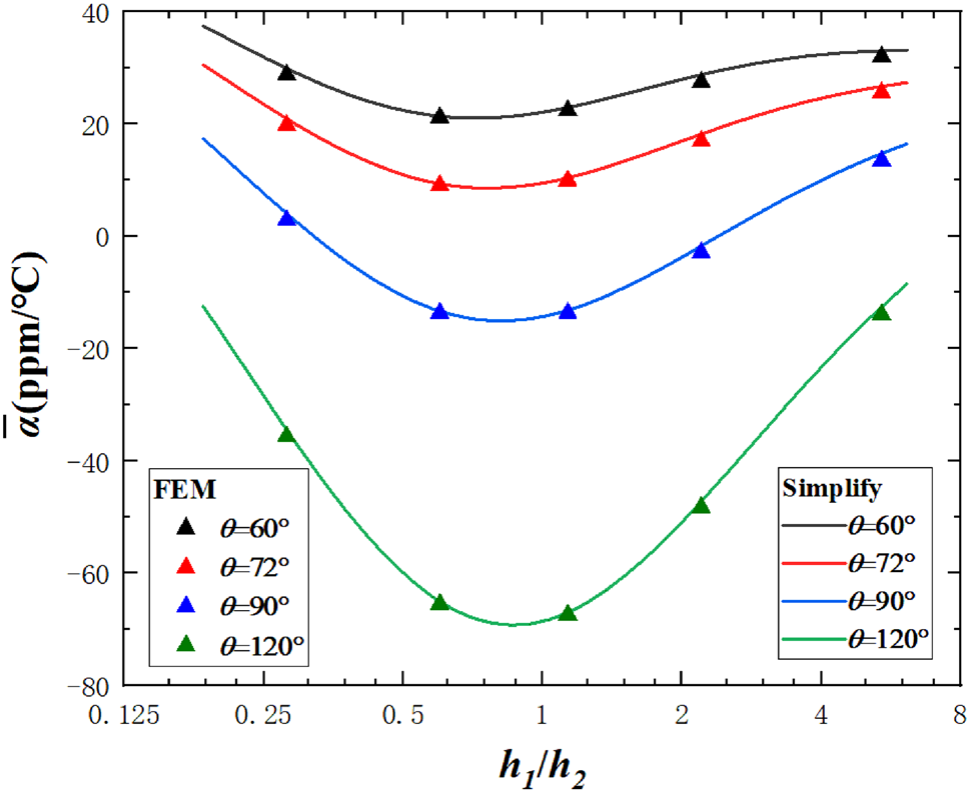
The effects of ${{h_{1} } \mathord{\left/ {\vphantom {{h_{1} } {h_{2} }}} \right. \kern-\nulldelimiterspace} {h_{2} }}$ on the equivalent CTE at ${h \mathord{\left/ {\vphantom {h b}} \right. \kern-\nulldelimiterspace} b} = {{1} \mathord{\left/ {\vphantom {{1} {5}}} \right. \kern-\nulldelimiterspace} {5}}$.

Also, the effects of the thickness ratio ${{h_{1} } \mathord{\left/ {\vphantom {{h_{1} } {h_{2} }}} \right. \kern-\nulldelimiterspace} {h_{2} }}$ of the two materials on the equivalent CTEs of the curved beam were investigated. Figure 5 shows $\overline{\alpha }$ versus ${{h_{1} } \mathord{\left/ {\vphantom {{h_{1} } {h_{2} }}} \right. \kern-\nulldelimiterspace} {h_{2} }}$ at ${h \mathord{\left/ {\vphantom {h b}} \right. \kern-\nulldelimiterspace} b} = {1 \mathord{\left/ {\vphantom {1 5}} \right. \kern-\nulldelimiterspace} 5}$. Each curve corresponds to a different $\theta$. As observed, $\overline{\alpha }$ decreases then increases as ${{h_{1} } \mathord{\left/ {\vphantom {{h_{1} } {h_{2} }}} \right. \kern-\nulldelimiterspace} {h_{2} }}$ increases. This phenomenon is due to fact that when there exists a large difference between the thicknesses of the two materials, the bi-material beam will be closer to the single-material beam which is PTE, and the NTE effect will not be good enough compared with the bi-material beam with a certain ratio of the thicknesses of the two materials. Therefore, to obtain the maximum NTE effect of bi-material curved beams, understanding how to obtain the minimum $\overline{\alpha }$ is necessary.

### Maximum NTE

**Figure 6:**
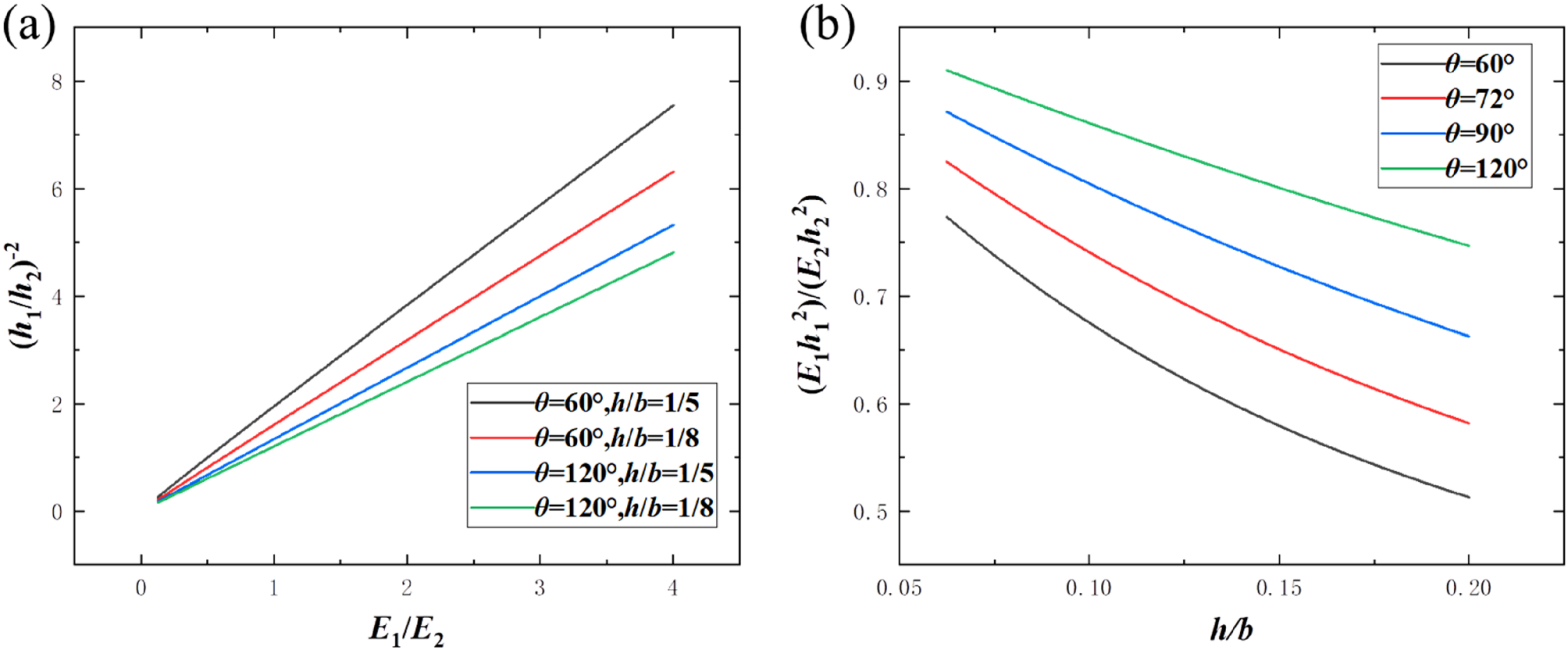
(**a**) Parameter relations at maximum NTE; (**b**) specific proportional coefficient curves corresponding to different ${h \mathord{\left/ {\vphantom {h b}} \right. \kern-\nulldelimiterspace} b}$ and $\theta$ at maximum NTE.

In this section, the relationships between ${{h_{1} } \mathord{\left/ {\vphantom {{h_{1} } {h_{2} }}} \right. \kern-\nulldelimiterspace} {h_{2} }}$ and other parameters are studied, in particular when the curved beam has the maximum NTE effect (minimum CTE). Take the derivative of Eq. (10) with respect to ${{h_{1} } \mathord{\left/ {\vphantom {{h_{1} } {h_{2} }}} \right. \kern-\nulldelimiterspace} {h_{2} }}$ and let the expression equal 0, the analytical relationships between ${{h_{1} } \mathord{\left/ {\vphantom {{h_{1} } {h_{2} }}} \right. \kern-\nulldelimiterspace} {h_{2} }}$ and other parameters when $\overline{\alpha }$ reaches the extreme value can be obtained. When $\overline{\alpha }$ reaches the extreme value, $\left( {{{h_{1} } \mathord{\left/ {\vphantom {{h_{1} } {h_{2} }}} \right. \kern-\nulldelimiterspace} {h_{2} }}} \right)^{ - 2}$ is approximately proportional to ${{E_{1} } \mathord{\left/ {\vphantom {{E_{1} } {E_{2} }}} \right. \kern-\nulldelimiterspace} {E_{2} }}$ and the proportional coefficient is not only dependent on ${h \mathord{\left/ {\vphantom {h b}} \right. \kern-\nulldelimiterspace} b}$ and $\theta$, but also independent from $\alpha_{{1}}$ and $\alpha_{{2}}$, as shown in Fig. 6a, which indicates that the ratio of static moments of the two layers should be specified to achieve maximum NTE. Figure 6b shows the curves of a specific proportional coefficient $\frac{{E_{1} h_{1}^{2} }}{{E_{2} h_{2}^{2} }}$ corresponding to different ${h \mathord{\left/ {\vphantom {h b}} \right. \kern-\nulldelimiterspace} b}$ and $\theta$ when maximum NTE effects are achieved, and these curves will become the guidance of the design of the maximum NTE of bi-material beams.

## Extensions of the analyses

### Extension of the analytical formula

**Figure 7:**
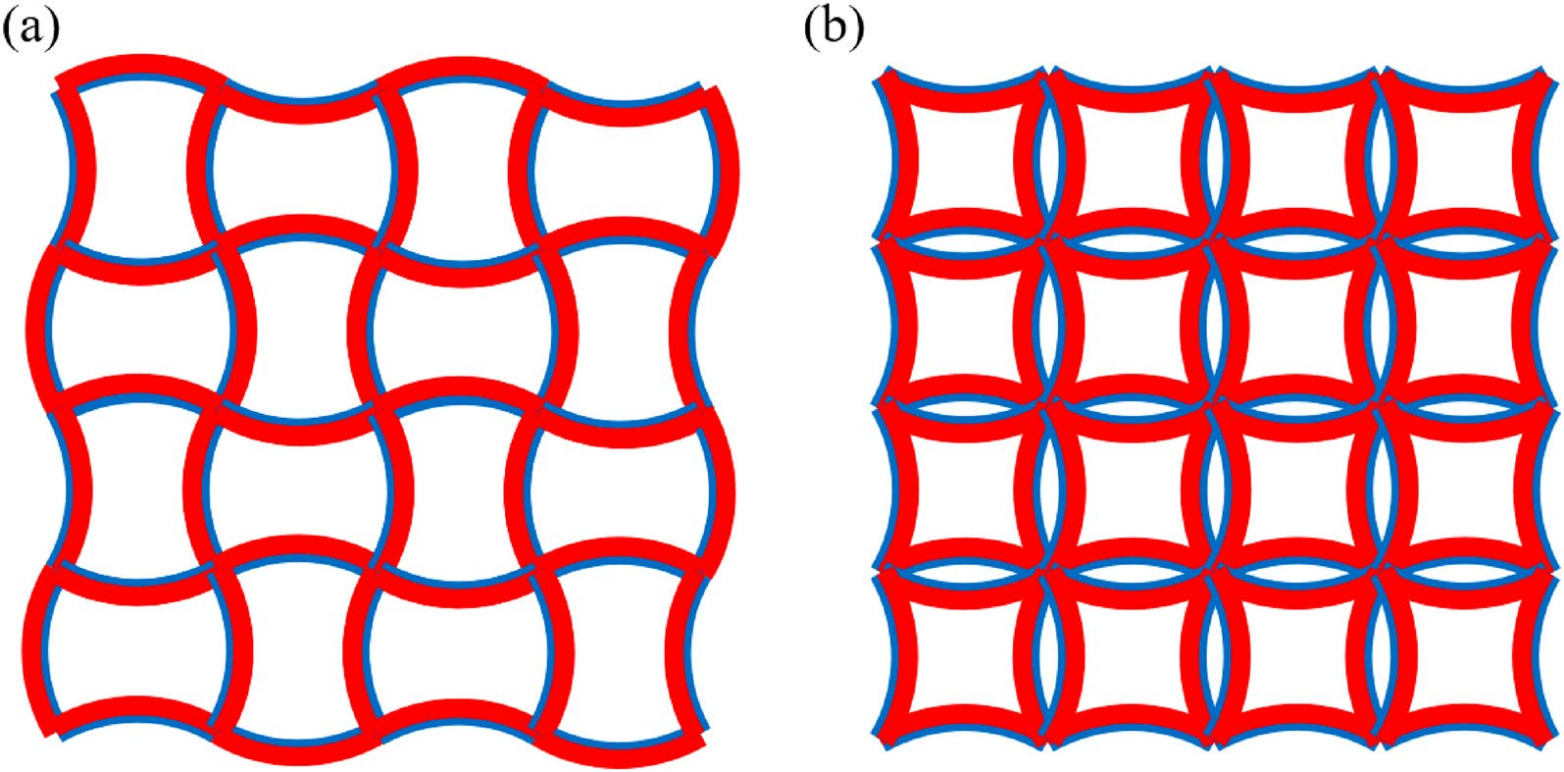
Two types of NTE metamaterials.

For the structure in Fig. 7a, there are no bending moments at both ends of the bi-material beams during thermal expansion. Both ends of the bi-material beam can be regarded as free boundaries according to the antisymmetry of the structure. Therefore, the proposed analytical formula can be used to calculate the equivalent CTE. However, for the structure in Fig. 7b, bending moments exist at both ends of the bi-material beam during thermal expansion according to the symmetry of the structure, in which case the third equation of Eq. (2) is not valid. However, modifying the formula derivation in “Pure bending of a single-layer curved beam under thermal–mechanical coupling” section can solve this problem.

According to the symmetry of Fig. 7b, the rotation angles at both ends of the beam are zero.

Therefore,

13 $$\frac{{\partial u_{\varphi } }}{\partial \rho } = 0$$

Substituting Eq. (1) into Eqs. (13) and (2) except the third equation, the following is obtained

14 $$\left\{ {\begin{array}{*{20}l} {A_{1} = \frac{{a^{2} b^{2} jE_{1} E_{2} \left( {\alpha_{1} - \alpha_{2} } \right)\Delta T}}{{b^{2} \left[ {b^{2} \left( {m - p} \right) + c^{2} \left( {q - m} \right)} \right] + a^{2} \left[ {b^{2} \left( {p - n} \right) + c^{2} \left( {n - q} \right)} \right]}}} \hfill \\ {B_{1} = 0} \hfill \\ {C_{1} = \frac{{ - b^{2} jE_{1} E_{2} \left( {\alpha_{1} - \alpha_{2} } \right)\Delta T}}{{b^{2} \left[ {b^{2} \left( {m - p} \right) + c^{2} \left( {q - m} \right)} \right] + a^{2} \left[ {b^{2} \left( {p - n} \right) + c^{2} \left( {n - q} \right)} \right]}}} \hfill \\ \end{array} } \right.$$

Substituting Eq. (14) into Eq. (7), the following can be obtained

15 $$\overline{\alpha } = \frac{{a^{2} \left[ {b^{2} \left( {\alpha_{1} p - \alpha_{2} n} \right) + c^{2} \left( {\alpha_{2} n - \alpha_{1} q} \right)} \right] + b^{2} \left[ {b^{2} \left( {\alpha_{2} m - \alpha_{1} p} \right) + c^{2} \left( {\alpha_{1} q - \alpha_{2} m} \right)} \right]}}{{b^{2} \left[ {b^{2} \left( {m - p} \right) + c^{2} \left( {q - m} \right)} \right] + a^{2} \left[ {b^{2} \left( {p - n} \right) + c^{2} \left( {n - q} \right)} \right]}}$$

If ${{h_{1} } \mathord{\left/ {\vphantom {{h_{1} } b}} \right. \kern-\nulldelimiterspace} b}$ and ${{h_{2} } \mathord{\left/ {\vphantom {{h_{2} } b}} \right. \kern-\nulldelimiterspace} b}$ are small, the following can be obtained

16 $$\overline{\alpha } = \frac{{\alpha_{2} \left[ {2\frac{b}{h} + 1 + 2\frac{{h_{1} }}{{h_{2} }}\left( {\frac{b}{h} - 1 - \mu_{1} } \right)} \right] + \alpha_{1} \frac{{E_{1} h_{1} }}{{E_{2} h_{2} }}\left[ {\frac{{h_{1} }}{{h_{2} }}\left( {2\frac{b}{h} - 1} \right) + 2\left( {\frac{b}{h} + 1 + \mu_{2} } \right)} \right]}}{{\left[ {2\frac{b}{h} + 1 + 2\frac{{h_{1} }}{{h_{2} }}\left( {\frac{b}{h} - 1 - \mu_{1} } \right)} \right] + \frac{{E_{1} h_{1} }}{{E_{2} h_{2} }}\left[ {\frac{{h_{1} }}{{h_{2} }}\left( {2\frac{b}{h} - 1} \right) + 2\left( {\frac{b}{h} + 1 + \mu_{2} } \right)} \right]}}$$

This is the equivalent CTE of a bi-material beam under constrained rotations at both ends and also the equivalent CTE of the structure in Fig. 7b.

### Nonlinear problems

The derivations of the above formulae are based on small deformation. For the finite deformation problem, the incremental theory can be used. Based on the updated Lagrangian description (ULD), the deformation of the first incremental step is obtained from the initial state, and then the deformation state of the first incremental step is used as a known condition to find the unknown quantities of the second incremental step, and so on until the final incremental step is done.

Assume that before the *i*th incremental step, the radius of curvature of the beam is $b_{i - 1}$ and the center angle is $\theta_{i - 1}$, then after the *i*th incremental step, the radius of curvature and the center angle of the beam become:

17 $$\left\{ {\begin{array}{*{20}l} {b_{i} = b_{i - 1} + \left. {u_{\rho } } \right|_{{\rho = b_{i - 1} }} } \hfill \\ {\quad = b_{i - 1} + \frac{1}{{E_{{1}} }}\left( { - \left( {1 + \mu_{{1}} } \right)\frac{{A_{{1}} }}{{b_{i - 1} }} - B_{1} b_{i - 1} + \left( {1 - \mu_{{1}} } \right)\left( {B_{{1}} \ln b_{i - 1} + C_{{1}} } \right)b_{i - 1} } \right) + \alpha_{{1}} \Delta T_{i} b_{i - 1} } \hfill \\ {\theta_{i} = \theta_{i - 1} + \frac{{\partial u_{\varphi } }}{\partial \rho } = \theta_{i - 1} + \frac{{2B_{{1}} \theta_{i - 1} }}{{E_{{1}} }}} \hfill \\ \end{array} } \right.$$

The coefficients $A_{1} , \, B_{1} , \, C_{1}$ are shown in Eq. (9). It is important to note that these coefficients contain *b*, and need to be in incremental forms, too.

The average CTE of the arc is:

18 $$\overline{\alpha }_{i} = \frac{{b_{i} \sin \left( {{{\theta_{i} } \mathord{\left/ {\vphantom {{\theta_{i} } 2}} \right. \kern-\nulldelimiterspace} 2}} \right) - b_{0} \sin \left( {{{\theta_{0} } \mathord{\left/ {\vphantom {{\theta_{0} } 2}} \right. \kern-\nulldelimiterspace} 2}} \right)}}{{b_{0} \sin \left( {{{\theta_{0} } \mathord{\left/ {\vphantom {{\theta_{0} } 2}} \right. \kern-\nulldelimiterspace} 2}} \right)\sum {\Delta T_{i} } }}$$

**Figure 8:**
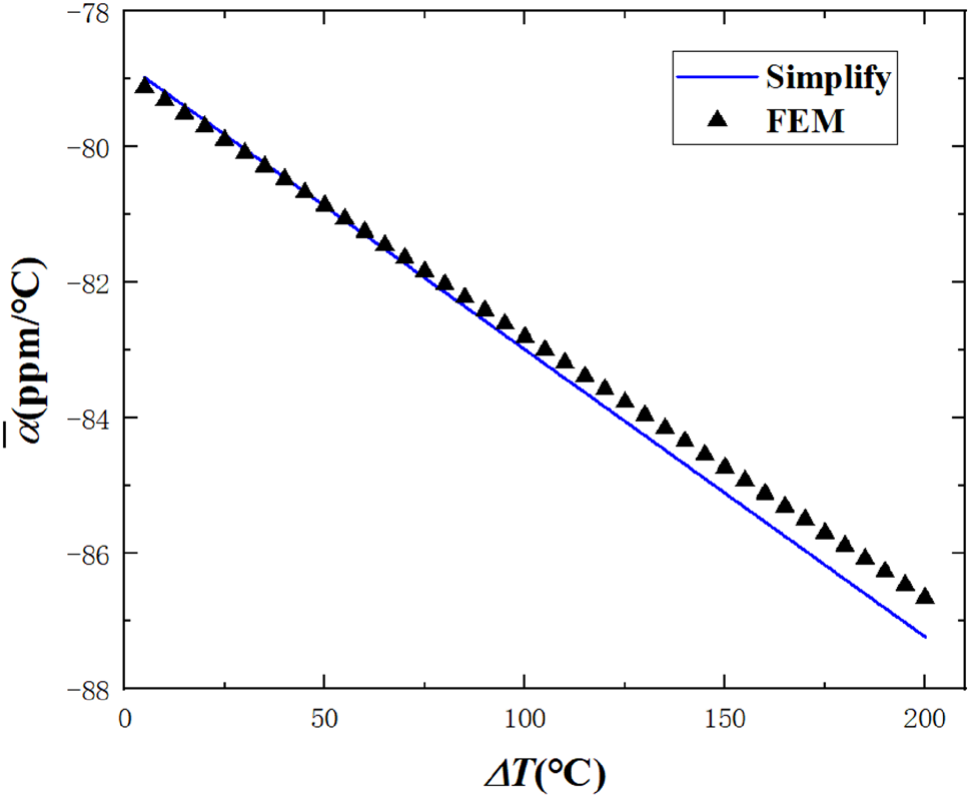
Comparison between theoretical formula and finite element method of nonlinear CTE.

Figure 8 shows the results of the comparison between Eq. (18) and the nonlinear finite element method. The geometric parameters of the beam and temperature increment step are $b_{0} = 80\;{\text{mm}}$, $\theta_{0} = 90^\circ$, $h_{1} = h_{2} = 4\;{\text{mm}}$, $T_{0} = 0\;^\circ {\text{C}}$, $\Delta T = 0.5\;^\circ {\text{C}}$. After the final incremental step, the radius of curvature of the beam is $b_{{{\text{final}}}} = 76.2\;{\text{mm}}$, and the central angle is $\theta_{{{\text{final}}}} = 95.4^\circ$. It can be seen that the results obtained by Eq. (18) are in good agreement with the finite element results, and the maximum error is only 0.66%, which shows that the analytical formula in this paper is also applicable to the incremental theory.

In addition, the incremental theory can also be applied to the range of material nonlinearity. Two kinds of materials with non-linear physical parameters, high chromium steel and austenitic stainless steel, whose average CTEs (compared with 20 °C) and elastic moduli are shown in Tables 1 and 2, are used for analyzing.

**Table 1:** Average CTEs at different temperatures (compared with 20 °C).

Temperature (°C)	0	50	100	150	200	250	300	350	400
High chromium steel (ppm/°C)	9.29	9.59	9.94	10.20	10.45	10.67	10.96	11.19	11.41
Austenitic stainless steel (ppm/°C)	16.28	16.54	16.84	17.06	17.25	17.42	17.61	17.79	17.99

**Table 2:** Elastic moduli at different temperatures.

Temperature (°C)	− 20	20	100	150	200	250	300	350	400
High chromium steel (GPa)	203	201	198	195	191	187	181	175	165
Austenitic stainless steel (GPa)	199	195	191	187	184	181	177	173	169

Besides, the physical parameters corresponding to the temperatures that are not given in the tables can be obtained by linear interpolation. Considering that the CTEs given in the tables are relative to the initial configuration, that is, based on total lagrangian description (TLD), it needs to be transformed into the CTEs based on ULD, and the transformation formula is as follows:

19 $$\alpha_{{{\text{ULD}}}} = \frac{{\ln \left( {1 + \alpha_{{{\text{TLD}}}} \Delta T} \right)}}{\Delta T}$$

In the iterative process, the CTE and elastic modulus corresponding to the temperature of the final state are substituted into Eq. (18) for iterative calculation. It should be noted that in this algorithm, to obtain the equivalent CTEs of the temperature corresponding to a certain iterative step, it is necessary to take the temperature as the final state and use the corresponding physical parameters for another set of iterative calculations.

**Figure 9:**
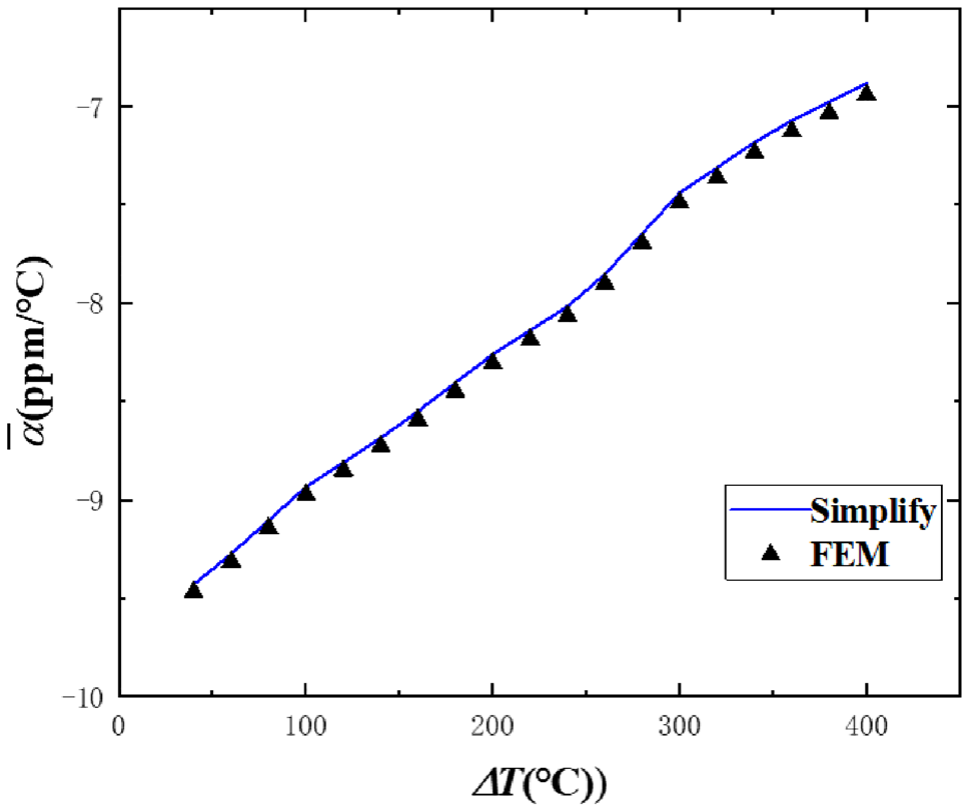
Comparison between theoretical formula and finite element method of nonlinear CTE.

Figure 9 shows the results of the comparison between Eq. (18) and the nonlinear finite element method. The geometric parameters of the beam and temperature increment step are $b_{0} = 80\;{\text{mm}}$, $\theta_{0} = 90^\circ$, $h_{1} = h_{2} = 4\;{\text{mm}}$, $T_{0} = 20\;^\circ {\text{C}}$, $\Delta T{ = 0}{\text{.5}}\;^\circ {\text{C}}$. After the final incremental step, the radius of curvature of the beam is $b_{{{\text{final}}}} = {77}{\text{.5}}\;{\text{mm}}$, and the central angle is $\theta_{{{\text{final}}}} = 93.24^\circ$. It can be seen that the results obtained by Eq. (18) are in good agreement with the finite element results, and the maximum error is only 0.8%, which shows again that the analytical formula in this paper is also applicable to the incremental theory.

## Novel NTE metamaterials

### Structure design

**Figure 10:**
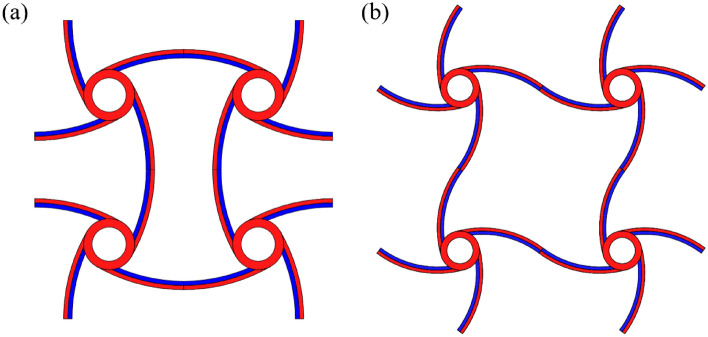
Two novel NTE metamaterials: (**a**) anti-tetra chiral structure; (**b**) tetra chiral structure.

As shown in Fig. 10, two novel NTE metamaterials are designed in this paper by replacing the straight ligaments in the tetra chiral/anti-tetra chiral honeycomb with bi-material curved beams, where the blue parts represent Material 1 (PI) in the inner layers of the curved beams, and the red parts represent Material 2 (PMMA) in the ring and the outer layers of the curved beams. In the chiral honeycomb, the ligaments are divided into two segments that bend in opposite directions. For both metamaterials, when the ambient temperature rises, the curvature of the ligaments will increase, making the rings closer to each other, which will approximately produce NTE effect on the whole. Besides, the bending of the bi-material beam during thermal deformation is the basis of realizing the NTE effect, which leads to the rotation of the rings in the chiral structure and the rotation amplifies the NTE effect of the whole structure. Therefore, the bi-material design makes more contribution to the NTE than chirality. In fact, for the chiral structure in Fig. 10b, when the circles shrink, the NTE effect will be weakened and when the circle shrinks to a point, the structure will degenerate into the structure in Fig. 7a, whose NTE effect is much weaker compared with the structure in Fig. 10b.

### The equivalent CTE

**Figure 11:**
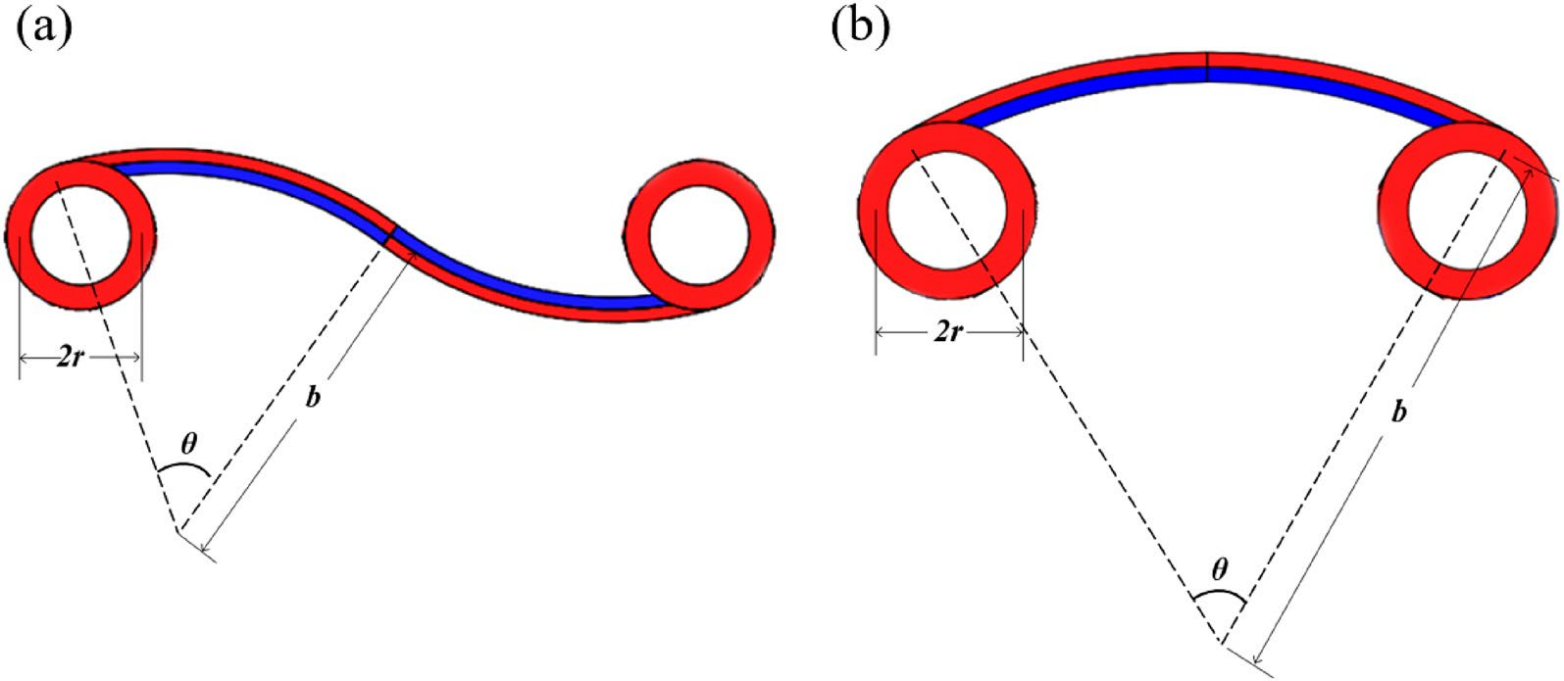
The geometry parameters of the novel metamaterials: (**a**) tetra chiral structure; (**b**) anti-tetra chiral structure.

First, the equivalent CTE of tetra chiral honeycomb is deduced. As can be seen in Fig. 11a, in the tetra chiral honeycomb, the radius of curvature of the ligament arc is $b$, the central angle is $\theta$, and the radius of the ring is $r$.

According to the geometrical relationship, the chord length of circular arc is:

20 $$l = 2b\sin \frac{\theta }{2}$$

and the distance between the two rings is:

21 $$L = 2\sqrt {\left( {r\cos \frac{\theta }{2}} \right)^{2} + \left( {l - r\sin \frac{\theta }{2}} \right)^{2} }$$

The change of the distance between the centers of the two rings after the temperature rises $\Delta T$ is:

22 $$\Delta L = 2\sqrt {\left[ {\left( {r + \Delta r} \right)\cos \left( {\frac{\theta + \Delta \theta }{2}} \right)} \right]^{2} + \left[ {\left( {l + \Delta l} \right) - \left( {r + \Delta r} \right)\sin \left( {\frac{\theta + \Delta \theta }{2}} \right)} \right]^{2} } - L$$

As can be known from “Derivation, simplification and verification of analytical formulas” section:

23 $$\left\{ {\begin{array}{*{20}l} {\mathop {\lim }\limits_{\Delta T \to 0} \frac{\Delta r}{{\Delta T}} = \alpha_{2} r} \hfill \\ {\mathop {\lim }\limits_{\Delta T \to 0} \frac{\Delta \theta }{{\Delta T}} = \frac{{\partial^{2} u_{\varphi } }}{\partial \rho \partial \Delta T} = \frac{{2B_{1} \theta }}{{E_{1} \Delta T}}} \hfill \\ {\mathop {\lim }\limits_{\Delta T \to 0} \frac{\Delta l}{{\Delta T}} = \overline{\alpha }l} \hfill \\ \end{array} } \right.$$

Therefore, the equivalent CTE of the tetra chiral honeycomb is:

24 $$\begin{aligned} \alpha_{{{\text{chiral}}}} & = \mathop {\lim }\limits_{\Delta T \to 0} \frac{\Delta L}{{L\Delta T}} \\ & = \mathop {\lim }\limits_{\Delta T \to 0} \frac{1}{L}\left( {\frac{\Delta L}{{\Delta r}}\frac{\Delta r}{{\Delta T}} + \frac{\Delta L}{{\Delta \varphi }}\frac{\Delta \varphi }{{\Delta T}} + \frac{\Delta L}{{\Delta l}}\frac{\Delta l}{{\Delta T}}} \right) \\ & = \frac{4}{{L^{2} }}\left[ {\alpha_{2} r^{2} + \overline{\alpha }l^{2} - rl\left( {\overline{\alpha }\sin \frac{\theta }{2} + \alpha_{2} \sin \frac{\theta }{2} + \cos \frac{\theta }{2}\frac{{B_{1} \theta }}{{E_{1} \Delta T}}} \right)} \right] \\ \end{aligned}$$

Then, the equivalent CTE of the anti-tetra chiral honeycomb is derived. As can be seen in Fig. 11b, in the anti-tetra chiral honeycomb, the radius of curvature of the semi-ligament arc is $b$, the central angle is $\theta$, and the radius of the ring is $r$. The difference between tetra and anti-tetra honeycomb lies in that the distance between the two rings in anti-tetra honeycomb is:

25 $$L = l - 2r\sin \frac{\theta }{2}$$

The equivalent CTE of the anti-tetra chiral honeycomb can then be derived using the same method:

26 $$\alpha_{{\text{anti - chiral}}} = \frac{1}{L}\left( {\overline{\alpha }l - 2\alpha_{2} r\sin \frac{\theta }{2} - 2r\cos \frac{\theta }{2}\frac{{B_{1} \theta }}{{E_{1} \Delta T}}} \right)$$

### Finite element model

The 8-node plane stress element CPS8R of the finite element software Abaqus is utilized to perform numerical simulation to verify the results derived in “Structure design” section. A single cell is used for analysis. The displacement loads in the y direction are applied to the midpoints of the cross section of the ends of the left and right ligaments of the structure, and the displacement loads in the x direction are applied to the midpoint of the ends of the upper and lower ligaments according to the antisymmetry. The temperature load of 1 °C is applied to the whole structure, and the equivalent CTE can then be obtained.

### Validity and parameter analysis

In this section, the change of CTEs of chiral/anti-chiral honeycombs as the curvature of the ligament changes is studied when the radius $r$ and distance $L$ of the rings remain unchanged. Meanwhile, the validity of the results has been verified by the comparison with the finite element results.

**Figure 12:**
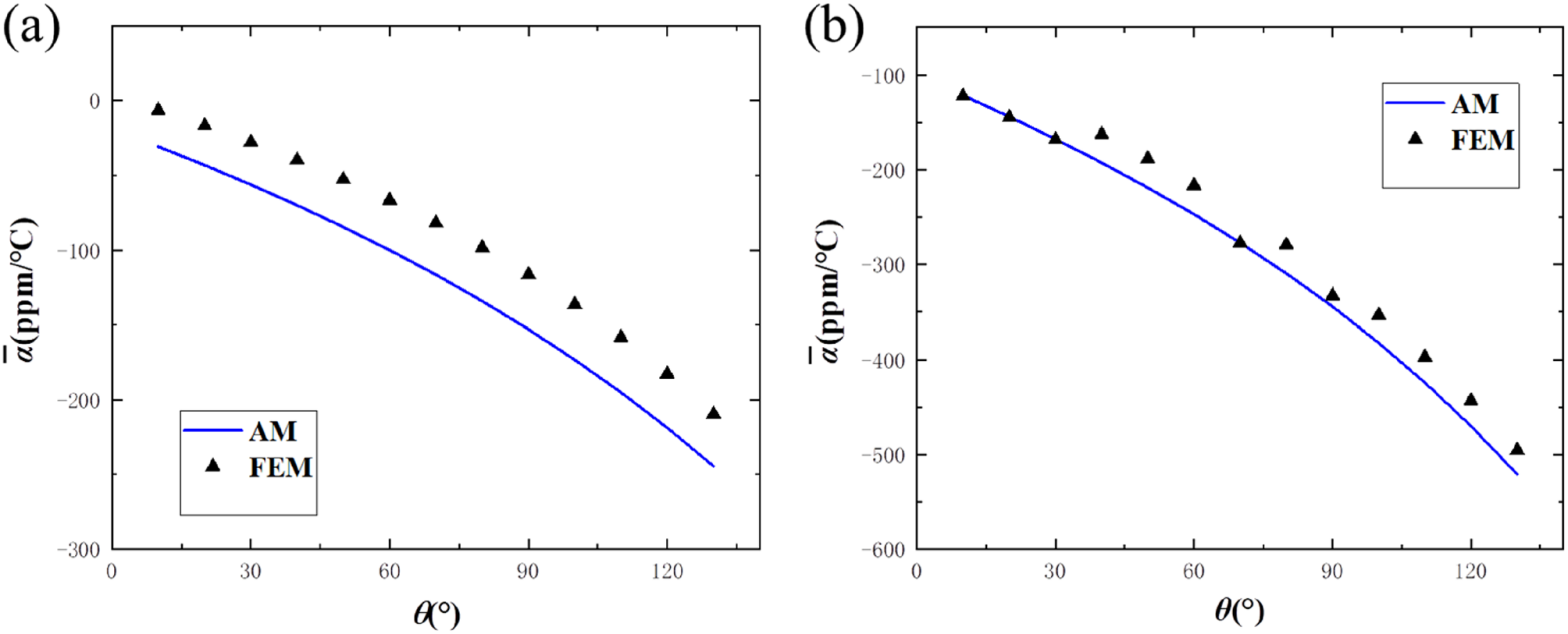
Comparison between theoretical formula and finite element method of the proposed metamaterials: (**a**) chiral structure; (**b**) anti-chiral structure.

As shown in Fig. 12, the analytical solutions are basically consistent with the finite element results, and the maximum relative error of chiral honeycomb does not exceed 9% while the maximum relative error of the anti-chiral honeycomb does not exceed 6%. With the increase of central angle, the NTE effects of both chiral and anti-chiral honeycombs increase, which means that the design method of replacing bi-material straight beams with curved beams can effectively increase the NTE effect of this kind of metamaterial. Besides, when the central angles are equal to 0, the structures will become the NTE structures proposed by Yu et al.^19^ and it is obvious that the proposed metamaterials in this paper could obtain higher NTE values compared with those according to Fig. 12. The jagged shape of the curve for FEM in Fig. 12b is due to the great robustness at the junction between the ring and the ligament and specific optimization problems need to be further studied. It should be noted that AM still means the results from analytical methods and FEM means the results from finite element methods in Fig. 12.

## Conclusion

In this study, the precise elasticity solution for the thermal expansion problem of bi-material curved beam is proposed based on the study in Ref^34^ and the simplified result is proposed based on the assumption that the slenderness ratio is small. This solves the problem that the accuracy of the formula in Ref^31^ at relatively large thicknesses (as compared to the radius) of the beam and small central angles are limited. The length of the simplified result is much shorter than that of the original result, and at the same time, it is still in great agreement with the numerical simulation result. Also, parameters and the nonlinear problems of the bi-material beam are analyzed based on the simplified formula during thermal deformation, and the conditions under which the bi-material circular beam achieves maximum NTE are identified. Finally, a class of novel NTE metamaterials are designed by introducing bi-material curved beams into tetra/anti-tetra chiral honeycomb, and the equivalent CTEs of this kind of metamaterials are given by using the simplified formula proposed in this paper. The proposed analytical formula can effectively analyze the thermal expansion of bi-material circular beams and has great potential for applications.

## Data Availability

The datasets generated and analysed during the current study are available from the corresponding author on reasonable request.
